# Patterns of Contagious Yawning and Itching Differ Amongst Adults With Autistic Traits vs. Psychopathic Traits

**DOI:** 10.3389/fpsyg.2021.645310

**Published:** 2021-04-09

**Authors:** Molly S. Helt, Taylor M. Sorensen, Rachel J. Scheub, Mira B. Nakhle, Anna C. Luddy

**Affiliations:** ^1^Departments of Psychology and Neuroscience, Trinity College, Hartford, CT, United States; ^2^New England Center for Children, Southborough, MA, United States; ^3^Albany Medical College, Albany, NY, United States; ^4^Department of Public Health, Boston University, Boston, MA, United States; ^5^Mass General Hospital, Boston, MA, United States

**Keywords:** contagion, autism, psychopathy, empathy, yawn

## Abstract

Both individuals with diagnosed with Autism Spectrum Disorder (ASD) and individuals high in psychopathic traits show reduced susceptibility to *contagious yawning;* that is, yawning after seeing or hearing another person yawn. Yet it is unclear whether the same underlying processes (e.g., reduced eye gaze) are responsible for the relationship between reduced contagion and these very different types of clinical traits. College Students (*n* = 97) watched videos of individuals yawning or scratching (a form of contagion not reliant on eye gaze for transmission) while their eye movements were tracked. They completed the Interpersonal Reactivity Index (IRI), the Autism-Spectrum Quotient (AQ), the Psychopathy Personality Inventory-Revised (PPI-R), and the Adolescent and Adult Sensory Processing Disorder Checklist. Both psychopathic traits and autistic traits showed an inverse relationship to contagious yawning, consistent with previous research. However, the relationship between autistic (but not psychopathic) traits and contagious yawning was moderated by eye gaze. Furthermore, participants high in autistic traits showed typical levels of contagious itching whereas adults high in psychopathic traits showed diminished itch contagion. Finally, only psychopathic traits were associated with lower overall levels of empathy. The findings imply that the underlying processes contributing to the disruptions in contagious yawning amongst individuals high in autistic vs. psychopathic traits are distinct. In contrast to adults high in psychopathic traits, diminished contagion may appear amongst people with high levels of autistic traits secondary to diminished attention to the faces of others, and in the absence of a background deficit in emotional empathy.

Both Autism Spectrum Disorder (ASD) and psychopathy have been described as empathy disorders (e.g., Platek et al., 2003; Schürmann et al., 2005), and previous research has shown that both individuals with ASD and individuals with high levels of psychopathic traits show diminished eye contact early in life (Robins et al., 2001; Dadds et al., 2014) as well as diminished susceptibility to contagious yawning (Helt et al., 2010; Rundle et al., 2015). At the same time, the clinical presentation of these two disorders is quite distinct, with individuals with ASD being thought to display deficits in cognitive empathy (imagining what someone else might be feeling based on knowing the facts of their situation) and individuals with psychopathy thought to display deficits in emotional empathy (internalizing a small part of the emotions of a person right in front of you, such as laughing more during a movie because the person next to you is laughing, or tearing up during a movie as you watch one of the character's tear up) (Jones et al., 2010). The ability to resonate with the inner states of those around you involves a number of separable processes that could potentially go awry in distinct ways (Frith, 2012). Contagion is a primitive form of empathy (Hatfield et al., 2009) that may lay a foundation for more complex forms of empathy (Prochazkova and Kret, 2017). Elucidating the mechanisms by which it deviates from typical development in these two clinical populations may, ultimately, inform early intervention theory and practice.

## Autism and Psychopathy: The “Empathy Disorders”

ASD and psychopathy have both been described as empathy disorders. Hoffman (2000) defines empathy as “any process where the attended perception of the object's state generates a state in the subject that is more applicable to the object's state or situation than to the subject's own prior state or situation.” Under such a broad definition of empathy, autism, and psychopathy do indeed both appear to be empathy disorders. Global empathy deficits have been reported in both groups (Baron-Cohen and Wheelwright, 2004; Ali et al., 2009; Dadds et al., 2009). Gillberg (1992) proposed the term “disorders of empathy” be used as a blanket term for all forms of autism because of the core feature of difficulty in reciprocal social interaction. Psychopathy, as identified in adults, has sometimes actually been defined as a lack of empathy (Frick et al., 1994). Both individuals with psychopathy and ASD share certain types of functional deficits such as difficulty recognizing and processing the emotional expressions of others (Hall et al., 2007; Clark et al., 2008), and neural activation patterns in both groups reveal reduced embodiment of the emotions of the target (Eigsti, 2013; Chen et al., 2017).

At the same time, the specific nature of the empathy deficits across these two groups differs sharply. Psychopathy is characterized by traits such as “cold-heartedness” (Hare, 1991; Blair et al., 2004; Weber et al., 2008; Frick and Viding, 2009). In contrast, Kanner's (1944) initial description of children with autism referred to “extreme…aloneness.” In other words, individuals with ASD tend to be described as “in their own world” as opposed to individuals with psychopathic traits who are more often described as tuned into the world around them but unfeeling toward those in it. The overarching construct of empathy can be and has been meaningfully divided into *cognitive* and *emotional* empathy (Nummenmaa et al., 2008; Shamay-Tsoory et al., 2009; Smith, 2010) and individuals with ASD and individuals with psychopathy show distinct patterns of strengths and deficits across these subtypes of empathy.

*Cognitive empathy* is characterized by the ability to take the perspective of another based on context (e.g., Shamay-Tsoory et al., 2009). A large body of research shows that individuals with Autism Spectrum Disorder (ASD) tend to struggle with cognitive empathy (see Andreou and Skrimpa, 2020 for a review); however, the regarding deficits in cognitive empathy amongst individuals with psychopathic traits are less consistent. Research has shown that individuals with ASD are less able to take the perspective of others; in other words, to understand the beliefs (Charman and Baron-Cohen, 1992) goals (Zalla et al., 2006), actions (Vivanti et al., 2011), and mental states (Happe, 1994) of others, and to understand how context affects emotions (Frith and Happé, 1994; Frith and Frith, 2006; Jones et al., 2010; Gaigg, 2012; Schwenck et al., 2012). In contrast, parents do not report deficits in cognitive empathy amongst children with callous and unemotional traits (the traits in childhood that often serve as a precursor to adult traits of psychopathy) (Pasalich et al., 2012), and multiple studies have shown intact cognitive empathy (and perspective-taking) in both children or adults with psychopathic traits (Blair, 1996; Richell et al., 2003; Dolan and Fullam, 2004; Anastassiou-Hadjicharalambous and Warden, 2008; Jones et al., 2010; Schwenck et al., 2012), with one study even suggesting heightened cognitive empathy in this population (Hansen et al., 2008).

*Emotional empathy* is characterized as an instinctive, or automatic, physical embodiment of someone else's emotions. At a broad level, both individuals with psychopathic traits and individuals with ASD tend to demonstrate diminished emotional empathy. Specifically, both individuals with high levels of psychopathic traits and individuals with ASD have been shown to display reduced physiological arousal to emotional stimuli (Aniskiewicz, 1979; Hare et al., 1991; Patrick et al., 1993; Blair, 1999; Jones et al., 2010; Marsh et al., 2011; de Wied et al., 2012), and reduced emotional contagion (Helt et al., 2010; Rundle et al., 2015; O'Nions et al., 2017).

Critically, each group also appears to show some islands of heightened response to the emotions of those around them, but these areas differ between groups. Individuals with high levels of psychopathy show enhanced cortical excitability when viewing others in pain, but indifference to other's distress. In contrast, individuals with ASD have typical spontaneous sensorimotor responses when viewing others in pain (Fan et al., 2014; Hadjikhani et al., 2014) and show appropriate physiological arousal to others' distress (Sigman et al., 1992; Blair, 1999). Among individuals with milder symptoms of ASD, facial EMG activity, evidence of facial mimicry, and emotional contagion, is heightened compared with controls in response to happy and fearful faces (Magnée et al., 2007). In contrast, individuals high in psychopathic traits tend to show reduced autonomic reactivity to the fear of others (Birbaumer et al., 2005; Marsh and Cardinale, 2012).

### Contagion and Empathy

Certain actions, such as yawning and scratching are “*contagious*,” meaning that they frequently result in automatic mimicry and thus propagate through a group (Hatfield et al., 1994). For example, seeing (Provine and Hamernik, 1986; Helt et al., 2019; Cordoni et al., 2021) or hearing a yawn (Massen et al., 2015; Helt et al., 2019) elicits yawning in observers ~30–60% of the time. Similarly, seeing others scratching elicits contagious itch in observers ~40–80% of the time. The behaviors that are most likely to be contagious are those that signify the *inner states* of others (Hatfield et al., 1994). Thus, contagion may reflect the *emotional* component of empathy in that it results in the observer being brought closer to the inner state of the target without necessarily evoking the *cognitive* component of empathy (e.g., newborns presumably are unable to identify that another baby is the source of the emotion when crying contagiously, and certainly cannot understand why).

Several studies have reported non-significant links between susceptibility to yawn contagion and scores on various empathy measures (Haker and Rössler, 2009; Bartholomew and Cirulli, 2014; Gottfried et al., 2015; see Massen and Gallup, 2017 for a review). However, other studies have suggested an association between contagious yawning and some facet of empathy (Platek et al., 2003; Arnott et al., 2009; Rundle et al., 2015). For example, susceptibility to contagious yawning is positively related to performance on cognitive empathy measures, such as Theory of mind tasks, and negatively related to schizotypal traits (Platek et al., 2003). In addition, contagious yawning tends to be greatest in response to kin, then friends, then acquaintances, and lastly strangers (Norscia and Palagi, 2011; Norscia et al., 2020)—a pattern consistent with other empathic behaviors (Preston and de-Waal, 2002, although Massen et al., 2015, reported that participants were no more likely to yawn contagiously to a member of their political “in” vs. “out” group). Furthermore, contagious yawning is not present in infants or toddlers (Helt et al., 2010) but rather develops during the preschool years (Cordoni et al., 2021) along the same timeline as other empathic abilities (Perner and Lang, 1999). For the purposes of the current study, the most important point may be that both individuals who are diagnosed with clinical conditions affecting empathy (e.g., schizophrenia) and individuals who merely show traits of clinical conditions affecting empathy (callous and unemotional traits, schizotypal traits) show reduced *spontaneous* susceptibility to contagious yawning (Platek et al., 2003; Haker and Rössler, 2009; Rundle et al., 2015) as well as other forms of contagion (e.g., Haker and Rössler, 2009; O'Nions et al., 2017).

### Contagion in Psychopathy and ASD

Participants with a clinical diagnosis of ASD are *less* susceptible than age-matched, typically developing peers to the contagious yawns of strangers when they are shown video recordings (Senju et al., 2007; Helt et al., 2019), played audio recordings (Giganti and Esposito Ziello, 2009), or during live interactions (Helt et al., 2010) of other people yawning. However, yawning contagion appears spared when stimuli are familiar loved ones (Helt et al., 2019), and when participants are explicitly cued to look at the eyes of the target (Senju et al., 2009; Usui et al., 2013; Helt et al., 2019). Indeed, Massen and Gallup (2017) propose that contagious yawning is more linked to visual attention than to empathy. In addition, children with ASD have been shown to be *more* susceptible to itch contagion, which is a form of contagion not transmitted *via* the eyes (Helt et al., 2020). Scambler et al. (2007) reported that when children with ASD paid attention to the emotional stimulus, they were just as likely to demonstrate an empathetic response. In other words, the extant literature is consistent with the hypothesis that individuals with ASD do not have inherent reduced susceptibility to contagion provided they are attending to the stimuli. In cases in which an individual with ASD attends to and correctly classifies the other's emotional state, emotional empathy should be intact.

In contrast, the limited research on this topic seems to indicate even in cases in which the target's emotional state has been correctly classified, the individual with psychopathic traits is still less likely to experience emotional contagion (Luckhurst et al., 2017) or to generate an empathic response (see Waller et al., 2020 for a review). Indeed, this lack of emotional empathy alongside intact cognitive empathy is proposed to enable those with extremely high levels of psychopathic traits to manipulate others and to account for a disproportionate number of crimes (Blair, 2005).

### Eye Gaze in Psychopathy and ASD

Although their ultimate clinical presentation is distinct, both researchers in the field of ASD (Dawson et al., 2004) and those in field of callous and unemotional traits (Dadds et al., 2008, 2012) have argued that an early failure in social attention may lead to cascading errors in the development of empathy. Eye contact between target and observer increases arousal, releases oxytocin, activates neural networks associated with social thinking (Becchio et al., 2007) and appears to be a crucial component in the process of empathic development. Eye gaze deficits appearing in childhood characterize the developmental trajectory of both ASD and psychopathic traits (Dadds et al., 2006; Jones and Klin, 2013).

#### Eye Gaze in Psychopathy

Children with callous and unemotional traits are reported to have displayed reduced eye contact with their caregiver during infancy (Dadds et al., 2011) and to display reduced eye gaze to target in experimental paradigms (Dadds et al., 2006). A hallmark characteristic of individuals with psychopathic traits is an impairment in the ability to recognize fear stimuli (Blair et al., 2004). However, when children with callous–unemotional traits are explicitly asked to attend to the eyes of fearful faces, they show normal levels of fear recognition (Dadds et al., 2008). Similarly, adults with psychopathic disorder often fail to show startle potentiation (e.g., Benning et al., 2005), though when their attention is explicitly directed to fear-relevant information, they show normalized fear-potentiated startle responses (Newman et al., 2010). Taken together, these studies suggest the possibility that some of the deficits in emotion empathy observed in individuals with psychopathic traits may be secondary to eye gaze avoidance.

#### Eye Gaze in ASD

Atypical eye contact is one of the first (Robins et al., 2001) and most significant symptoms of autism, and the range of contexts in which eye gaze is normal amongst individuals with ASD tends to be limited compared to neurotypical peers. For example, children with ASD have been shown to have reduced activation of the fusiform face area when viewing the faces of strangers (Schultz et al., 2000), but not when viewing the faces of their parents (Pierce et al., 2004) or when explicitly instructed to attend to the eye region of the target (Hadjikhani et al., 2004). Similarly, a small number of studies have shown that individuals with ASD demonstrate typical contagious yawning either when explicitly asked to attend to the eyes of the target (Senju et al., 2009; Usui et al., 2013; Helt et al., 2019) or when the target stimuli consist of their parents (Helt and Fein, 2016; Helt et al., 2019).

### Current Study

Across both groups, observed empathy deficits appear to be largely associated with a failure to direct attention to stimuli that normally elicit emotional response. At the same time, accounts abound of individuals with ASD showing empathic behaviors when they are attending and understand the context, which is not the case for individuals with callous and unemotional, or psychopathic, traits. It is unclear whether these two clinical groups begin with similar starting states (diminished eye gaze resulting in a failure of further social learning and experience) and branch off from one another in terms of trajectory after the development of contagion, or whether, even in the acquisition of early forms of empathy such as contagion, the underlying mechanisms at play are already quite distinct.

The current study explored the extent to which susceptibility to contagious yawning may be differentially moderated by eye contact in individuals with high and low levels of ASD and psychopathic traits in a non-clinical population. In order to do this, we used eye tracking to measure the eye gaze to the eyes of the stimuli targets for the contagious yawning trials and employed a form of contagion not reliant on eye gaze (itching) as a comparison condition. We hypothesized that the mechanisms underlying reduced contagion differ between these groups, with reduced contagion being secondary to reduced social attention in the high ASD traits group only (reflecting “aloneness”—social signals are often missed, but when they are not, contagion functions typically) and independent of eye gaze in the psychopathy trait group (reflecting “aloofness”—even when social signals are processed, contagion malfunctions). We further hypothesized that self-reported autistic traits would be negatively correlated with self-reported cognitive empathy, and that self-reported psychopathic traits would be negatively correlated with self-reported emotional empathy.

## Methods

### Participants

Participants were 100 Trinity College students (50 female, 47 male), ages 18–23, recruited through psychology and neuroscience courses. The sole exclusion criteria was corrected-to-normal vision and no eyeglasses for easier calibration to the eye tracker; as such it is possible that some participants may have met clinical criteria for ASD or Antisocial Personality Disorder or other clinical conditions. Three participants were excluded due to loss of eye tracking data or inadequate visual attention, resulting in a final sample of 97. See Table 1 for participant data.

**Table 1:** Participant characterization variables.

Chronological age (years)	21.48 (1.93); 18.75–24.58
AQ scores	16.08 (7.01); 4–34
PPI-R scores	49.08 (12.54); 27–88
IRI scores	67.5 (15.3); 24–96
Percentage of time looking at eyes across conditions	13.5 (8.8) 1.7–46.8

### Measures

*The Autism-Spectrum Quotient* (Baron-Cohen et al., 2001). The AQ is a 50 item, forced choice format questionnaire used to quantify Autism Spectrum Disorder traits is adolescents and adults of at least average intelligence. In the current study, the Cronbach alpha coefficient was 0.89.

*The Psychopathic Personality Inventory-Revised* (Lilienfeld and Widows, 2005). The PPIR is a self-report assessment of psychopathic traits in non-criminal populations. In the current study, the Cronbach alpha coefficient was 0.81.

*Interpersonal Reactivity Index* (Davis, 1980). The IRI is a multidimensional, self-report measure of empathy comprised of 28-items answered on a 5-point Likert scale ranging from “Does not describe me well” to “Describes me very well” with half of the items reverse scored. The IRI comprises four subscales: Perspective Taking (the tendency to spontaneously adopt the psychological point of view of others, for example, “I try to look at everybody's side of a disagreement before I make a decision”), Fantasy (respondents' tendencies to transpose themselves imaginatively into the feelings and actions of fictitious characters in books, movies, and plays, for example, “After seeing a play or movie, I have felt as though I were one of the characters”), Empathetic Concern (“other-oriented” feelings of sympathy and concern for unfortunate others, for example, “I often have tender, concerned feelings for people less fortunate than me”), and Personal Distress (“self-oriented” feelings of personal anxiety and unease in tense interpersonal settings, for example, “Being in a tense emotional situation scares me”). In the current study, the Cronbach alpha coefficient was 0.78.

### Apparatus

**Figure 1:**
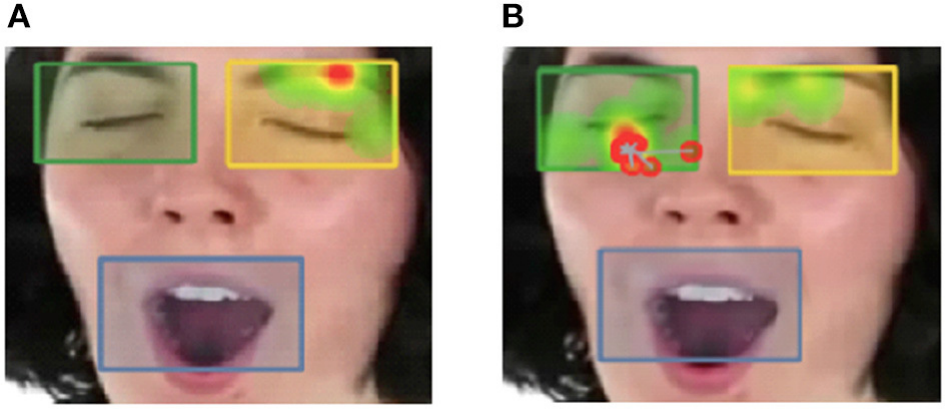
Sample differences of visual fixation points for **(A)** individuals high in autistic traits, and **(B)** individuals high in psychopathic traits. Gradients of most attended areas on the heat maps are shown from red to yellow to green. Areas of interest were drawn around the eyes and mouth. Percentage of time that participant eye gaze was fixated within the eye region boxes shown below was positively correlated with tendency to yawn contagiously.

Participants' visual gaze fixation patterns were captured using an Applied Science Laboratories (ASL) Desktop Eye Tracker. Before participants began viewing the yawn and itch stimuli they were calibrated to the eye tracker. One computer was used to present stimuli videos. The eye tracking unit was positioned at the base of the stimulus computer monitor to collect and analyze pupil/corneal reflection points of the participant (the center of the participant's pupil and a reflection from the surface of the cornea) at a sampling rate of 120 Hz per second, and was connected to the computer presenting the stimuli. “Areas of Interest” were specified around the eye region of the stimuli as well as around the mouth region (see Figure 1) and percentage of total time spent on each AOI by stimulus condition (yawn or itch) was used for analysis. Fixation durations for each AOI by condition were summed across all scenes within each condition, to create total fixation duration variables for each AOI type. An AOI that covered the entire screen for each trial was also created, in order to measure overall visual attention or any loss of eye tracking data. This was not used in analysis but was used to exclude two participants. Adjacent fixations were merged, with the maximum time between merged fixations set to 75 ms and the maximum angle between merged fixations set to 0.5°. Merging fixations close in time and proximity prevents longer fixations from being separated into shorter fixations because of data loss or noise. Fixations shorter than 30 ms that did not meet criteria for merging were discarded.

### Stimuli

**Figure 2:**
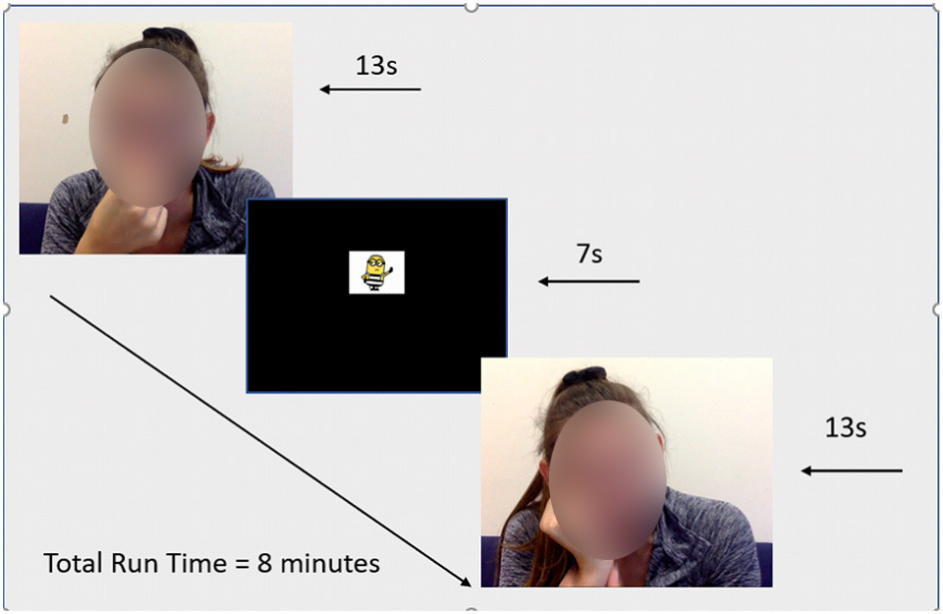
Sample stimuli.

The stimuli were created by having 11 adults wear wireless earbuds while simultaneously watching youtube clips of others yawning and scratching and looking into our video camera. Thus, the videos of yawns and scratches (both under weak voluntary control; see Provine, 2005) were themselves contagious yawns and itches/scratches. In half of the stimulus clips, the adults are looking at the camera, and in half their eyes are averted. Itches and yawns judged to be genuine by the “actor” and the experimenter were used in a pilot study with undergraduates to ensure their contagious properties, and any clips that did not produce contagious responses in the pilot group were discarded. The original yawning and itching videos from youtube used to create the remainder of the stimuli bank were also included (*n* = 2), for a final set of 24 clips; 12 yawning, 12 itching. Each of the 24 video clips contained 1 yawn (M = 13 s each) or 1 bout of scratching (M = 13 s each) with fixation cartoons shown at eye level between contagious stimuli clips within each block, lasting ~7 s, for a total of 8 min of viewing time (see Figure 2).

### Procedure

The clips were presented by computer, and participants' contagious yawn or itch responses were recorded *via* a video camera mounted to the top of the 23.6-inch monitor in order to capture the participants' faces and upper bodies, as they sat 18 inches from the monitor and speakers. Audio level was preset, but participants were given the option to adjust the volume during the video introduction. Participants were told that they would be viewing a series of faces making different expressions. The order of the video clips was randomized for each participant. After viewing the videos, participants completed the paper and pencil measures. Participants were tested between 10 a.m. and 3 p.m. Each participant was consented prior to starting the experiment and debriefed afterwards. This study was approved by Trinity College's Institutional Review Board.

### Data Coding and Analysis

Each video was independently coded by two different raters who were naïve to participant condition (i.e., whether the participant they were coding was watching yawns or itches as target behaviors were being coded). Coding criteria for full yawn included the following physical manifestations: open mouth, narrow eyes, and indrawn breath (Provine, 2005). Inter-rater reliability was 100% for full yawns (two partially concealed yawns were excluded from data analysis). Coding criteria for itch required observation of the participants' hand moving to a body location and scratching (increased “nose twitching” was observed during the itch trials—it was not coded but we wonder if there may be merit in exploring this in future studies). Yawning and scratching behaviors were coded both during *congruent* conditions (i.e., yawning coded during presentation of yawning stimuli and itching coded during the presentation of itching stimuli) and during *incongruent* conditions (e.g., participant yawning coded during presentation of itching stimuli and participant scratching coded during the presentation of yawning stimuli). Overall, there was a low rate of scratching during the yawn videos (*n* = 4 itches in total across all 97 participants vs. *n* = 43 scratches across all participants during itch videos) and a low rate of yawning during the scratching videos (*n* = 8 yawns during non-yawn stimuli vs. *n* = 51 in yawn condition), indicating that the stimuli were successful in eliciting contagious responses. Contagion susceptibility was operationalized as the frequency of the target behavior throughout the entire set of stimuli.

Participant eye gaze patterns while watching stimuli videos were recorded using the ASL desktop eye tracker and analyzed with ASL results plus software. The eyes and mouths of individuals in the stimuli videos were defined as the areas of interest in ASL. Percentage of time participant eye gaze fell within the eyes AOI was calculated separately for yawn stimuli and itch stimuli trials.

## Results

Because the mean number of yawns per participant (M = 0.31) and itches per participant (M = 0.29) were both <1, participants were coded dichotomously as “contagious yawners” or not and “contagious itchers” or not. Overall, 31% (30/97) of the sample displayed at least one contagious yawn during the presentation of yawn stimuli and 29% (28/97) of the sample displayed at least one contagious itch during the presentation of itch stimuli. Table 1 describes the Mean, SD, and range of the sample in terms of chronological age, eye gaze, and measures of empathy, autistic traits, and psychopathic traits.

### Contagious Yawning

Total scores on the IRI (empathy), AQ (autistic traits), and the PPI-R (psychopathic traits) were entered into a binary (participants were coded as contagious yawners or not) logistic regression model with contagious yawning serving as the dependent variable. The Hosmer-Lemeshow Goodness of Fit Test was non-significant, supporting the model's validity, $\chi_{\left(8\right)}^{2}=$ 9.9, *p* = 0.25. The overall model, containing all predictor variables was significant, $\chi_{\left(3\right)}^{2}=$ 28.1, *p* < 0.001, explaining between 25.1% (Cox and Snell R square) and 35.5% (Nagelkerke R squared) of the variance in contagious yawning response. However, only AQ score (measure of autistic traits) and PPI-R score (measure of psychopathic traits) made unique statistically significant contributions to the model. High levels of psychopathic traits were found to negatively predict contagious yawning $\chi_{\left(3\right)}^{2}=$ −14.9, *p* < 0.001. In other words, the higher participants rated themselves in psychopathic traits, the less likely they were to yawn contagiously. Second, high levels of autistic traits also negatively predicted contagious yawning $\chi_{\left(1\right)}^{2}=$ −12.6, *p* < 0.001, indicating that, overall, the higher participants rated themselves in autistic traits, the less likely they were to yawn contagiously than those who rated themselves lower in ASD traits. These over-arching results largely replicate previous research indicating that contagious yawning is diminished, under naturalistic conditions, amongst individuals with so-called “empathy disorders.” Total empathy scores were *not* unique predictors of contagious yawning status, $\chi_{\left(1\right)}^{2}=$ 0.07, *p* = 0.68.

In order to test the hypothesis that the diminished tendency for people higher in autistic (but not psychopathic) traits to yawn contagiously was moderated by diminished eye gaze to target, the percentage of total time each participant spent fixated on the eyes of the yawning stimulus targets was calculated and this was entered into the regression model with AQ scores and PPI-R scores. Adding percent fixation on the eyes of the target yawn stimuli to the model increased the total variance explained by the model to 44.4%, *F*_(3, 93)_ = 14.19, *p* = 0.002. When percentage of eye gaze to target was added to the model, the relationship between AQ scores and contagious yawning decreased in strength [going from $\chi_{\left(1\right)}^{2}=$ 12.3, *p* < 0.001, to $\chi_{\left(1\right)}^{2}=$ 8.1, *p* = 0.003], indicating that higher ASD scores predicting lower contagious yawning was moderated by the association between high ASD scores and lower percentage of time looking at the eyes. In other words, at least some of the reason for lower contagious yawning amongst participants with higher AQ scores can be explained by the tendency of participants with higher AQ scores to spend less time looking at the eyes of the target during the yawning stimulus trials. Notably, entering eye gaze information into the model slightly increased the strength of the relationship between psychopathic traits and contagious yawning [going from $\chi_{\left(1\right)}^{2}=$ −14.6, *p* < 0.001 to $\chi_{\left(1\right)}^{2}=$ −16.3, *p* < 0.001], indicating that the lower rates of contagious yawning among those with higher psychopathic traits *cannot* be explained by differences in eye gaze patterns toward the target. Entering attention to the mouth region (AOI) into the model, did not significantly change the model, $\chi_{\left(1\right)}^{2}=$ 28.4, *p* = 0.002.

Finally, the subscales of the empathy scale (personal distress, empathetic concern, fantasy, and perspective taking) were entered into the model, in order to determine whether either cognitive (perspective taking, fantasy) or emotional (personal distress, empathetic concern) aspects of empathy might show a differential relationship with overall contagion. The model remained largely unchanged, $\chi_{\left(1\right)}^{2}=$ 29.6, *p* = 0.02 with PPI = R scores, AQ scores, and eye gaze, remaining significant predictors.

### Contagious Itching

Total scores on the IRI, AQ, and the PPI-R were entered into a binary logistic regression model with contagious itching (it occurred or did not) as the dependent variable. The model was significant, $\chi_{\left(1\right)}^{2}=$ 23.23, *p* < 0.001, explaining between 22% (Cox and Snell R square) and 31.5% (Nagelkerke R square) of the variance, but only because psychopathic traits inversely predicted contagious itch, $\chi_{\left(1\right)}^{2}=$ −12.99, *p* < 0.001. Neither autistic traits, $\chi_{\left(1\right)}^{2}=$ −0.214, *p* < 0.64, nor empathy, $\chi_{\left(1\right)}^{2}=$ −1.55, *p* < 0.21, demonstrated a significant relationship to contagious itch. Next, percent of total fixation time on the eyes of the itch stimulus targets was entered into the model leaving it largely unchanged, $\chi_{\left(1\right)}^{2}=$ 23.96, *p* < 0.001, and contributing no unique variance to the model, $\chi_{\left(1\right)}^{2}=$ 0.66, *p* = 0.45, indicating that direct eye gaze is not critical for transmitting contagious itch, as it is for transmitting contagious yawn. Finally, the subscales of the empathy scale (personal distress, empathetic concern, fantasy, and perspective taking) were entered into the model, $\chi_{\left(1\right)}^{2}=$ 33.986, *p* < 0.01, accounting for between 29.9 and 42.9% of the variance in contagious itch response. Both higher self-reported levels of personal distress, $\chi_{\left(1\right)}^{2}=$ 6.62, *p* = 0.01, and higher self-reported levels of psychopathic traits, $\chi_{\left(1\right)}^{2}=$ 9.70, *p* < 0.01, were associated with higher levels of contagious itch.

### Correlations

Spearman's rho is reported as it is more robust to non-normal distributions. Despite the fact that yawning and itching appeared to reflect the influence of distinct predictors, these behaviors showed a trend toward correlation, ρ = 0.161, *p* < 0.08 meaning that those who exhibited one contagious behavior were more likely to exhibit the other. AQ and PPI-R scores were inversely correlated, ρ = −0.449, *p* < 0.001, indicating that these are largely non-overlapping traits, and, indeed, AQ and PPI-R traits show distinct patterns of relationships to eye gaze and the empathy subscales of the IRI.

ASD traits showed an inverse correlation with percentage of eye gaze to the AOI encompassing the target's eyes across both yawn stimuli, ρ = −0.198, *p* = 0.05, and itching stimuli, ρ = −0.218, *p* = 0.05. Results thus suggest that individuals with higher levels of ASD traits tended to spend *less* time looking at the eye region of the target than people with lower levels of these traits. Levels of psychopathic traits were unrelated to the amount of time a participant spent looking toward the eye region of the target either during yawn trials, ρ = 0.102, *p* = 0.32, or itch trials, ρ = 0.051, *p* = 0.62.

**Figure 3:**
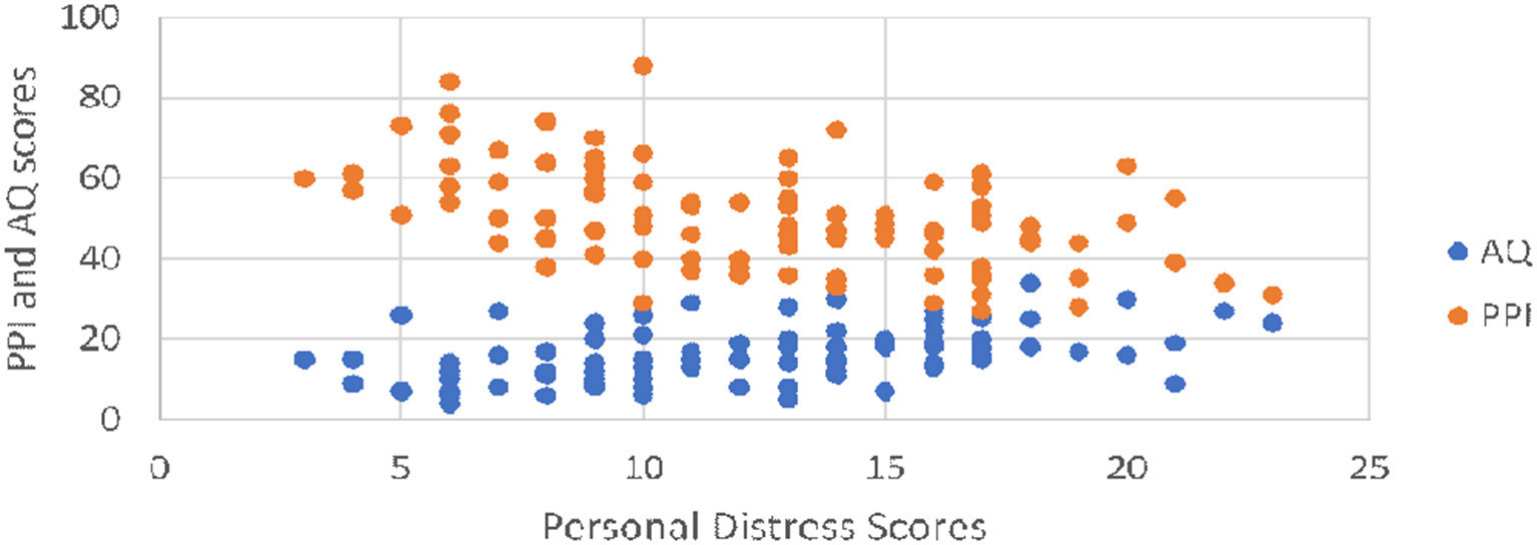
As AQ scores increased, so did personal distress. AS PPI-R scores increased, personal distress scores decreased.

Finally, ASD traits and psychopathic traits showed different relationships to different facets of empathy (as measured by the subscales of the IRI). The IRI is composed of 2 scales thought to measure cognitive empathy (Perspective Taking and Fantasy) and 2 scales thought to comprise emotional empathy Davis (1983, 1994): Empathetic Concern (the tendency to experience other-oriented feelings of sympathy and compassion in response to another's misfortune), and Personal Distress (the tendency to experience self-oriented feelings of discomfort and anxiety in response to another's misfortune). We had hypothesized that higher levels of autistic traits might be inversely associated with perspective taking and fantasy skills (aspects of cognitive empathy), but neither empathetic concern nor personal distress (aspects of emotional empathy). We predicted the reverse pattern for higher psychopathic traits; that they would be inversely related to emotional empathy subscales (empathetic concern and personal distress) and unrelated to cognitive empathy subscales (perspective taking and fantasy). These hypotheses were largely unsupported. ASD traits showed no relationship to perspective taking, ρ = −0.025, *p* = 0.81, fantasy, ρ = 0.075, *p* = 0.47, empathetic concern ρ = −0.110, *p* = 0.29, or overall empathy, ρ = 0.141, *p* = 0.20. However, they showed a significant *positive* relationship with personal distress, ρ = 0.504, *p* < 0.01 (see Figure 3). Psychopathic traits also did not have a significant relationship with perspective taking, ρ = −0.033, *p* = 0.75, empathetic concern, ρ = −0.158, *p* = 0.13, or fantasy ρ = −0.113, *p* = 0.28, and showed a *negative* relationship with overall empathy, ρ = −0.304, *p* < 0.01, as well as with personal distress, ρ = −0.488, *p* < 0.01 (see Figure 3).

## Discussion

Consistent with previous research showing that individuals with diagnoses of ASD (Helt et al., 2010) or high levels of psychopathic traits (Rundle et al., 2015), are less likely to yawn contagiously, the current study found that individuals with either high levels of ASD traits or high levels of psychopathic traits were less susceptible to contagious yawning than their peers low in both types of traits. However, the current study also extends previous results by suggesting that underlying mechanisms influencing this diminished contagion in individuals with these two different types of traits, appear to be distinct. Individuals high in psychopathic traits are less susceptible to both contagious yawn and contagious itch and this susceptibility appears to be unrelated to direct eye gaze, and to be negatively correlated with overall levels of empathy. Meanwhile, individuals high in autistic traits are only less susceptible to contagious yawning, and (unlike in individuals with high levels of psychopathic traits), this relationship is moderated by eye gaze toward the target. Autistic and psychopathic traits appear to have distinct empathy profiles, most notably, opposite relationships to *personal distress* (Figure 3). Overall, this pattern of results indicates that participants with high levels of autistic traits and high levels of psychopathic traits are largely non-overlapping groups who show distinct patterns of susceptibility to contagion.

First of all, in the current study, the relationship between autistic traits and contagious yawning was moderated by eye gaze. In other words, people with higher levels of autistic traits spent less time looking directly at the eyes of the stimuli targets, and that explained some of the variance in why they were less likely to yawn contagiously. In contrast, participants with higher levels of psychopathic traits were less likely to yawn contagiously despite no diminished tendency to attend to the eyes of the targets (see Table 2).

**Table 2:** Correlation matrix using spearman Rho.

	**Yawn**	**Itch**	**AQ**	**PPI-R**
Yawn		0.161 *P* = 0.08	^*^–0.211 *P* = 0.04	^*^–0.229 *P* = 0.05
Itch	0.161 *P* = 0.08		0.152 *P* = 0.14	^*^–0.447 *P* < 0.01
AQ	^*^–0.211 *P* = 0.04	0.152 *P* = 0.14		^**^–0.449 *P* < 0.01
PPI-R	^*^–0.229 *P* = 0.05	^**^–0.447 *P* < 0.001	^**^–0.449 *P* < 0.001	
Eye fixation to Itch stimuli only	0.185 *P* = 0.09	−0.040 *P* = 0.69	^*^–0.218 *P* = 0.05	0.051 *P* = 0.62
Eye fixation to Yawn stimuli only	^*^0.213 *P* = 0.05		^*^–0.198 *P* = 0.05	0.102 *P* = 0.32
IRI (total empathy score)	0.198 *P* = 0.08	0.177 *P* = 0.09	0.141 *P* = 0.20	^**^–0.304 *P* = 0.003
Empathetic concern	−0.027 *P* = 0.79	−0.051 *P* = 0.63	−0.110 *P* = 0.29	−0.158 *P* = 0.13
Fantasy	0.025 *P* = 0.80	0.090 *P* = 0.39	0.075 *P* = 0.47	−0.113 *P* = 0.28
Perspective taking	−0.019 *P* = 0.86	0.113 *P* = 0.28	−0.025 *P* = 0.81	−0.033 *P* = 0.75
Personal distress	−0.091 *P* = 0.38	^*^0.242 *P* = 0.02	^**^0.504 *P* < 0.01	^**^–0.488 *P* < 0.01

* *Correlation is significant, p < 0.05*.

** *Correlation is significant, p < 0.01*.

Furthermore, being high in autistic traits was unrelated to one's tendency to itch contagiously; in other words, individuals high in autistic traits were no more or less likely to demonstrate contagious itch. In contrast, participants high in psychopathic traits were significantly less likely to itch contagiously. In other words, individuals with high levels of autistic traits were only less likely to exhibit the form of contagion transmitted *via* eye gaze, whereas individuals with high levels of psychopathic traits were less likely to exhibit both forms of contagion.

Contagious itch and contagious yawn also showed distinct predictors. Observer eye gaze to eye region of target was not a positive predictor of contagious itching, indicating that contagious itch is not transmitted *via* eye gaze, as hypothesized. Observer eye gaze was a positive predictor of contagious yawning, consistent with previous research demonstrating that eye gaze is a significant step in the transmission of contagious yawning (Provine, 1989; Senju et al., 2009). High levels of “personal distress” predicted contagious itch only. Personal distress has been suggested to be a pure measure of (or proxy for) emotional contagion (the tendency to take on the inner states of others without necessarily showing awareness of the source of the emotional state) (Decety and Ickes, 2011), but it may be more accurately characterized as the tendency to take on the negative emotions of others. Importantly, autistic traits showed a positive relationship with *personal distress* (a sub-component of emotional empathy), and no relationship with overall empathy scores. In contrast, psychopathic traits showed a negative relationship with personal distress, as well as an inverse relationship with overall empathy scores.

**Figure 4:**
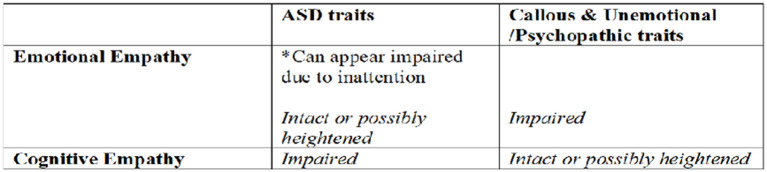
Theoretical Model of distinct types of empathy deficits amongst those with distinct clinical traits.

Overall results are consistent with the hypothesis that emotional empathy amongst individuals high in ASD traits is preserved (or possibly even heightened) under some conditions, and when “deficits” are found, they are likely secondary to diminished social attention. Although we must be cautious in speculating the extent to which a trait study such as this applies to individuals with diagnosed ASD, these results are consistent with a growing body of literature demonstrating that if an individual with ASD attends to and properly classifies an emotional or bodily signal associated with the inner state of another, they are likely to experience contagion and achieve bodily resonance with the other (e.g., Bird et al., 2010; Lockwood et al., 2013) (see Figure 4).

One previous study found that children with ASD showed *heightened* contagious itch compared with typically developing controls (Helt et al., 2020). The current study sheds light on a possible factor driving that relationship. The Adolescent and Adult Sensory processing checklist showed no relationship to the tendency to itch contagiously. Instead, the tendency to itch contagiously was found to be predicted by levels of *personal distress*. Similar to children diagnosed with ASD (Dziobek et al., 2008), participants high in ASD traits in the present study reported more personal distress (though no more contagious itch) than those low in ASD traits. Aversion to eye gaze, and the opportunities for shared emotion with others that accompany it (such as susceptibility to contagious yawning), increases along with ASD traits in the general population (Nummenmaa et al., 2012; Seara-Cardoso et al., 2012). Current results indicate that personal distress may do so as well. The positive relationship between “personal distress” (the tendency to take on the negative emotions of those around you) and autistic traits raises the possibility that young children with ASD may turn their attention inward as a defense mechanism against a tendency for heightened personal distress (as we would avert our eyes from something upsetting on a screen in order to self-regulate and reduce our arousal).

In contrast, diminished contagion amongst people with high levels of psychopathic traits does indeed appear to occur against the background of global deficits in emotional empathy. Dadds et al. (2006) reported that reduced eye gaze reverses fear contagion in children with callous and unemotional traits. However, the current results indicate that reduced eye gaze is not a viable contributing factor to why adults high in psychopathic traits are less likely to yawn (or itch) contagiously. Reduced eye gaze may be a signifier, rather than a cause, of reduced contagion in children with callous and unemotional traits, which is perhaps why increasing shared eye gaze between children with callous and unemotional traits and their caregivers have not been shown to have any sustained benefit (Dadds et al., 2019). In contrast, increasing eye gaze is often the first target in early intervention with young children with autism as it is so beneficial (e.g., Weisberg and Jones, 2019).

It is notable that high psychopathic traits (in opposition to autistic traits) were inversely related to personal distress. Perhaps young children with callous and unemotional traits (the developmental precursor of psychopathic traits) do not experience as much distress themselves and so they are unable to achieve bodily resonance with others even when they are attending to others. It seems possible that when an individual high in psychopathic traits is observing another's distress, the individual's own level of distress is low and the individual's tendency to take on the emotion of the target is low. Indeed, one study reported that levels of alexithymia, rather than levels of ASD traits, correlate with empathy deficits (Bird et al., 2010), supporting the possibility that being able to *feel* corresponding emotions with others early in life may be even more crucial to the affective components of empathic development than *attending* to the emotions of others.

The current results contribute to previous literature suggesting that those high in psychopathic traits may register the relevant features of the stimulus or target and the empathy system engages but malfunctions—for example when those with psychopathy experience pleasure at another's pain (Yochelson and Samenow, 1976). In contrast, those high in ASD traits may tend not to register the salient features of strangers or stimuli of strangers, and thus often fail to engage the empathy system with strangers, but, for example, may register family members, and some types of stimuli, normally; in other words, the system is intact but less often activated (Lombardo et al., 2007; Dziobek et al., 2008; Minio-Paluello et al., 2009; Frith, 2012; Lockwood et al., 2013).

### Limitations

Several factors limit interpretation of the current data. First of all, this was a relatively small and homogeneous sample of college students, not a clinical sample. However, as evidence has increased for a smooth continuum of ASD traits across the typical and clinical populations, as opposed to a discrete set of symptoms that appear only in cluster at a certain point of severity, research linking ASD traits to behavioral outcomes has become an increasingly common research focus (Happé and Frith, 2020). Previous studies have linked higher (subclinical) AQ scores to behavioral markers common in individuals diagnosed with ASD, from eye gaze (Nummenmaa et al., 2012; Seara-Cardoso et al., 2012) to more accurate pitch and temporal processing (Stewart et al., 2018) to lower performance on tests of social cognition and executive function (Gokcen et al., 2016). Second, the current study did not employ additional methods such as EMG, GSR, or fMRI during data collection. Third, all measures were self-report and no traits were verified by clinical observation. Future research in our lab will aim to address these limitations.

### Clinical Implications

Frith (2012) first suggested the approach of making “fine cuts” to distinguish the empathy deficits amongst those with autistic vs. callous and unemotional traits in order to further understand the neural and behavioral underpinnings of each disorder and how to best treat them. The current study's results support the hypothesis that affective impairments found in people with ASD are mainly related to the recognition and processing of incoming stimuli, rather than to the actual ability to feel emotional distress or concern. It is important to continue to gather data regarding this broader hypothesis, as it may inform aspects of interventions that aim to improve empathic functioning in individuals with ASD vs. psychopathy. For example, if evidence continues to accumulate to support distinctions in the types of empathy experienced by individuals with these different traits, it implies that interventions in autism should seek to increase the extent to which the individual identifies with, or assigns personal significance to, unfamiliar others as well as increasing the individual's comfort with eye gaze early in development. In contrast, these distinctions imply that attending to the eye gaze of others may not be a sufficient intervention for those with psychopathic traits, and that, perhaps instead, interventions should take a form of training in which the *experience* of emotion is linked to the expression of that emotion in others to increase the likelihood of shared emotional experience.

## Data Availability Statement

The raw data supporting the conclusions of this article will be made available by the authors, without undue reservation.

## Ethics Statement

The studies involving human participants were reviewed and approved by Institutional Review Board, Trinity College. The patients/participants provided their written informed consent to participate in this study. Written informed consent was obtained from the individual(s) for the publication of any potentially identifiable images or data included in this article.

## Author Contributions

MH: conceptualization, writing original first draft, editing, and supervision. RS: visualization, methodology, software, investigation, formal analysis, review, and editing. TS: methodology, software, investigation, formal analysis, review, and editing. MN: software, formal analysis, review, and editing. AL: software, investigation, and formal analysis. All authors: contributed to the article and approved the submitted version.

## Conflict of Interest

The authors declare that the research was conducted in the absence of any commercial or financial relationships that could be construed as a potential conflict of interest.
